# NF-κB-Mediated Upregulation of Tissue Factor Contributes to the Procoagulant Phenotype of Smooth Muscle Cells from Abdominal Aorta Aneurysm in Human

**DOI:** 10.1055/a-2665-2510

**Published:** 2025-08-12

**Authors:** Veronique Regnault, Melusine Didelot, Veronique Ollivier, Cecile Lakomy, Jeremy Lagrange, Huguette Louis, Cecile V. Denis, Serguei Malikov, Patrick Lacolley, Jean-Baptiste Michel

**Affiliations:** 1 Université de Lorraine, Inserm, DCAC, Nancy, France; 2 Inserm UMR_S 1148, CHU X. Bichat, Paris, France; 3 Université Paris-Saclay, Inserm, HITh, Le Kremlin-Bicêtre, France; 4 CHRU de Nancy, 54000 Nancy, France; 5 Department of Vascular and Endovascular Surgery, Nancy University Hospital, Vandoeuvre-lès-Nancy, France

**Keywords:** aneurysm, smooth muscle cell, tissue factor, thrombin

## Abstract

**Background:**

Aneurysms of the thoracic (TAA) and abdominal aorta (AAA) have different pathophysiological mechanisms. AAA has an intraluminal thrombus, while TAA does not. This suggests a prothrombotic phenotype in AAA, probably at the level of vascular smooth muscle cells (SMCs) known to express tissue factor (TF).

**Objectives:**

To explore the TF-dependent thrombin generation in SMCs in AAA compared with TAA and healthy aorta (HA) and the underlying mechanisms contributing to a procoagulant phenotype.

**Methods:**

Human HA, AAA, or TAA tissues and corresponding SMC primary cultures were used to analyze SMC-supported thrombin generation and TF expression.

**Results:**

In the absence of added TF, thrombin generation was increased at the surface of SMCs from AAA compared with TAA and HA, indicating a cellular procoagulant phenotype, which is transmitted through mitosis. Phosphatidylserine exposure was increased at the surface of SMCs from AAA. As expected, reactive oxygen species generation and the proinflammatory cytokine TNF-α were increased in SMCs from AAA. Overexpression of protease-activated receptor 2 and nuclear translocation of NF-κB p65 in SMCs and tissue from AAA triggered increased TF gene expression. Higher active basal TF expression was also observed in SMCs from AAA, which was inhibited by BAY 11–7082 (pharmacological inhibitor of IκK) and GB83 (pharmacological inhibitor of PAR-2).

**Conclusion:**

We demonstrated a PAR-2-mediated activation of the canonical NF-κB pathway, which triggers TF transcription in AAA. This procoagulant profile is transmitted from tissue to primary SMC cultures and through numerous passages, which can maintain thrombus formation.

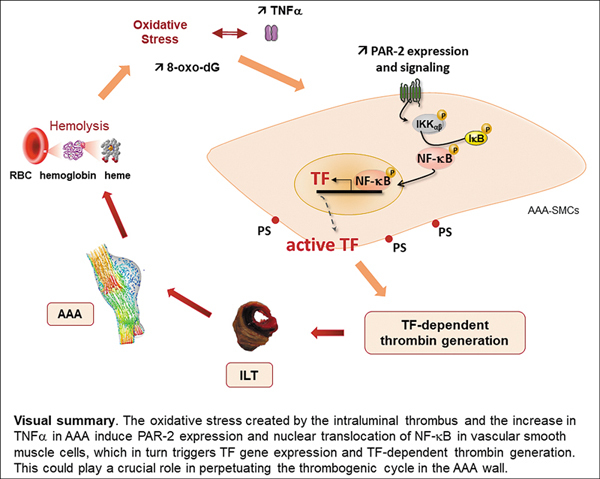

## Introduction

Aneurysms of the abdominal aorta (AAA) and of the ascending aorta (TAA) are both localized dilations of the human aorta, but are quite different in terms of their etiologies, pathophysiology, and local hemodynamics. An increase in wall permeability, leading to plasma insudation in the arterial wall, is a common characteristic of all aortic aneurysms. 1 2 AAA is a specific form of acquired atherothrombotic disease, 3 involving the dynamics (thrombin formation and fibrinolysis) of an intraluminal thrombus (ILT). Peroxidases released from red blood cells and neutrophils entrapped in the ILT trigger an important localized hemolytic-dependent oxidative stress. 4 5 Additionally, the expression level of TNF-α, a potent inflammatory mediator known to elicit tissue factor (TF) upregulation on vascular endothelium, is increased in human AAA tissue and is involved in aneurysm development. 6 7 8 Due to the presence of ILT, the AAA wall is usually devoid of healthy endothelial cells, exposing smooth muscle cells (SMCs) to blood. 3 In this context, SMCs, which constitutively express TF, could play an active role in thrombus formation in AAA. Indeed, beyond the loss of the anticoagulant properties of endothelial cells and the wall convection of prothrombin from blood, endothelial denudation in AAA tissue promotes direct contact between blood coagulation factors and SMCs. 9 Several teams have demonstrated the ability of SMCs to support TF-induced thrombin generation. 10 11 12 In addition, the oxidative stress created by ILT can increase TF gene expression and regulate its activity positively by posttranslational mechanisms. Thus, the prothrombotic phenotype in AAA may be related to the high synthesis of TF by SMCs, which in turn triggers excessive thrombin generation at the surface of SMCs. In contrast, TAA is not an atherothrombotic disease, but a disease involving SMC and extracellular matrix dysfunctions without ILT. Endothelium remains present in TAA, suggesting that anticoagulant pathways remain efficient.

Therefore, our working hypothesis is that SMCs derived from AAA could have a procoagulant phenotype compared with SMCs derived from healthy aorta (HA) and TAA. This procoagulant phenotype could explain the formation of ILT and its sustainable character in the AAA wall. To verify this, we analyzed thrombin generation initiated by SMC-expressed TF, and we explored the involvement of protease-activated receptor (PAR)-induced signaling.

## Methods

### Human Tissues

Human aortic tissues were obtained from the Inserm human CV biobank (BB-0033–00029), included in the European network BBMRI-ERIC, in accordance with French regular and ethical rules (DC2018–3141) and with the principles of the declaration of Helsinki. This biobank has already been used in several studies. 13 14 HA were collected from deceased organ donors with the authorization of the French Biomedical Agency (ABM, PFS09–007). AAA (RESAA study) and TAA tissues were sampled from patients undergoing surgery. The protocol was approved by the local ethics committee (CPPRB Paris-Cochin, approval n°2095 for AAA, and n°050432, Ambroise Paré, Boulogne, France for TAA). All patients with AAA or TAA provided written informed consent. The adventitial layer was immediately removed by macroscopic dissection in all aortic tissues; ILT was removed specifically from AAA. There are no ILTs in TAA and HA. Therefore, only intima + media were studied, with SMCs being the main cellular component in the media. HA were sampled at the thoracic aorta ( *n* = 7) or abdominal aorta ( *n* = 11) and were obtained from 10 women and 8 men, while AAA and TTA samples were collected from 14 men and 4 women ( Supplementary Table S1 , available in the online version only). Samples of these aortic tissues were, respectively, frozen, incubated to obtain tissue-conditioned media, fixed in paraformaldehyde for histology, or enzymatically digested for obtaining primary cultures of human SMCs.

### Tissue-Conditioned Media

The medial layer was cut into small pieces, which were incubated for 24 hours at 37°C in a standardized volume (6 mL/g of wet tissue) of RPMI 1,640 supplemented with antibiotics and antimycotics. The tissue-conditioned media were then collected and centrifuged, and the supernatant was aliquoted and frozen at −80°C until use.

### SMC Cultures

Primary cultures of healthy or aneurysmal human SMCs were obtained as reported previously. 15 After removal of the adventitial layer, ILT in AAA and washing, the media was submitted to enzymatic digestion in a mixture of 0.3% collagenase (LifeTechnologies, Gif-sur-Yvette, France) and 0.1% elastase (Worthington Biochemical Corporation, Lakewood, New Jersey, United States) for 3 hours at 37°C. Cells were cultured in SmGM-2 Smooth Muscle Growth Medium-2 Bulletkit (Lonza, Basel, Switzerland). SMCs were used between passages three and five. SMCs were seeded at a density of 7,500 cells/well into flat-bottom 96-well cell culture plates (MICROTEST 96) for 24 hours and washed with HEPES-buffered saline (HBS, 20 mM HEPES, 140 mM NaCl, pH 7.35 containing 5 g/L bovine serum albumin, BSA) before use.

For indicated experiments, cells were preincubated with the PAR-1 antagonist SCH79797 (Sigma–Aldrich) at 80 nM for 1 hour, the PAR-2 antagonist GB83 (Axon Medchem, Groningen, The Netherlands) at 10 nM for 4 hours or the inhibitor of NF-κB activation BAY 11–7082 (Abcam) at 10 nM for 4 hours or 10 µM for 1 hour.

### Thrombin Generation Assay

Venous blood from healthy donors, with informed consent in accordance with the guidelines of the EFS Grand Est (EFS LR54901/2023), was drawn into 0.106 M sodium citrate (9:1 vol/vol) containing vials. Normal pooled platelet-free plasma (PFP) was prepared by sequential centrifugation (190 g for 10 minutes, 1,750 g for 10 minutes, and 13,000 g for 30 minutes), aliquoted, and frozen at −80°C until use.

Calibrated automated thrombography was performed at 37°C in a microtiter plate fluorometer (Fluoroskan Ascent, ThermoLabsystems, Helsinki, Finland) using a dedicated software program (Thrombinoscope BV, Maastricht, The Netherlands) as reported previously. 10 16 Thrombin generation was triggered by recalcification of PFP added to washed SMC monolayers, which provide TF. 11 Thrombin generation curves were recorded in triplicate. Several parameters were derived from the thrombin generation curve, including lag time (time to thrombin burst) and peak (the maximum amount of thrombin formed), and the endogenous thrombin potential (ETP) was calculated as the area under the curve.

Some experiments were performed by replacing PFP with HBS or plasma deficient in factor VII, factor X, or prothrombin.

### Phospholipid Procoagulant Activity

Phospholipid-related procoagulant activity (PPA) at the surface of SMCs or in tissue-conditioned media was measured using a chromogenic assay based on prothrombinase activity, independently of TF-mediated activation of the coagulation, as described previously. 10 Briefly, washed adherent cells incubated with 50 µL of Tris buffered saline (TBS)-BSA or tissue-conditioned media were mixed with 50 µL of FXa (1.2 nM), FVa (2.4 nM), CaCl _2_ (15 mM), and 50 µL of a mixture of bovine prothrombin (6 µM) and Z-Gly-Gly-Arg-AMC substrate (1.25 mM) in 20 mM HEPES pH 7.5 containing 60 g/L BSA. The plate was placed in a Fluoroskan Ascent fluorometer and allowed to warm to 37°C for 5 minutes before kinetic readings over 30 minutes.

Phospholipid concentration was estimated from the initial rate of thrombin formation by reference to a standard curve constructed with a mixture of phosphatidylserine (PS), phosphatidylethanolamine, and phosphatidylcholine (20:20:60 mol%) and expressed as PS equivalents.

### Tissue Factor Quantification

Total RNA was extracted from SMCs using the RNeasy Mini kit (Qiagen, Les Ulis, France). First-strand cDNA was synthesized according to the manufacturer's instructions (Fermentas; Thermo Fisher Scientific, Waltham, Massachusetts, United States). Quantitative real-time polymerase chain reaction (RT-PCR) analysis was then performed using SYBR green PCR technology (Bio-Rad, Hercules, California, United States) using the following conditions: 10 seconds at 95°C; 45 seconds at the annealing temperature; 60 seconds at 72°C, repeated for 40 cycles. For each target gene, results from three independent RT-PCR analyses were expressed relative to GAPDH and ribosomal protein S29 (RPS29) expressions. The sequences of primers are given in Supplementary Table S3 (available in the online version only). The relative mRNA expression of the target gene was quantiﬁed by the ΔCt method using TaqMan Assay-on-Demand Hs99999905_m1.

TF antigen was quantified using the Human Coagulation Factor III/TF Quantikine ELISA (R&D Systems).

TF activity was determined using the Actichrome TF assay (American Diagnostica, Stamford, Connecticut, United States). Washed adherent SMCs overlaid with 75 µL of assay buffer to measure cell surface TF activity, or tissue-conditioned media (75 µL) were incubated with a mixture containing 25 µL of human factor VIIa, 25 µL of human factor X, and 25 µL of assay buffer from the kit. After 15 minutes at 37°C, factor X activation was stopped by adding 25 µL of 25 mM EDTA. Then, factor Xa activity was monitored after incubation with 25 µL of Spectrozyme FXa by measuring the absorbance at 405 nm every 15 seconds for 20 minutes at 37°C.

### ELISA

Factor X and factor VII were quantified in tissue-conditioned media by an immunoenzymatic method using the ZYMUTEST Factor X and ZYMUTEST Factor VII kits (Hyphen Biomed, Neuville-sur-Oise, France). Prothrombin concentration was quantified using the human coagulation factor II/thrombin ELISA Kit (Novus Biologicals, Abingdon, United Kingdom). TF pathway inhibitor (TFPI) was measured in cell culture lysates and supernatants using the Asserachrom total TFPI assay (Diagnostica Stago, Asnières sur Seine, France). Thrombomodulin was quantified using the Quantikine human thrombomodulin immunoassay (R&D Systems). Extracellular 8-oxo-7,8-dihydro-2′-deoxyguanosine (8-oxo-dG) was measured in cell culture supernatants using the HT 8-oxo-dG ELISA Kit II assay (R&D Systems).

### Histology, Immunohistochemistry, and Immunofluorescence

Samples of aortic tissues were fixed with 4% (w/v) buffered formaldehyde solution. Paraffin-embedded tissue sections were directly stained with Masson's trichrome or placed in citrate buffer pH 6.0 in a microwave to unmask antigens. Endogenous peroxidases were quenched with 1% H _2_ O _2_ , and nonspecific binding was blocked with 5% BSA for 1 hour at room temperature. Serial 5 µm sections were incubated overnight at 4°C with rabbit polyclonal anti-human fibrinogen (Abcam). HRP-conjugated anti-rabbit or anti-mouse IgG was used as a secondary antibody ( Supplementary Table S2 , available in the online version only). Binding was revealed using DAB technology.

Tissue sections treated for antigen retrieval or SMCs grown on glass coverslips and fixed with 4% (w/v) buffered formaldehyde solution were permeabilized with 0.1% Triton X-100 (Sigma–Aldrich) in phosphate-buffered saline (PBS). Nonspecific binding was blocked with 5% BSA for 1 hour at room temperature. Tissue sections or SMCs were incubated either overnight at 4°C with the primary antibodies ( Supplementary Table S2 , available in the online version only). Alexa Fluor 488-labeled anti-rabbit IgG and Alexa Fluor 555-labeled anti-mouse IgG were used as secondary antibodies, and nuclei were counterstained with 4',6'-diamidino-2-phenylindole (DAPI). Slides were visualized using an Eclipse CI-S microscope.

### Western Blotting

SMCs were harvested, washed twice with PBS, and homogenized in a cold lysis buffer (Roche Life Sciences, Meylan, France) containing a cocktail of proteases and phosphatase inhibitors. The lysates were clarified by centrifugation, and the protein concentration in the collected supernatants was measured using a Bradford Protein assay kit (Bio-Rad, Marnes-la-Coquette, France). Proteins were separated by 10% SDS-polyacrylamide gels under nonreducing conditions and then electro-transferred onto a nitrocellulose membrane. After blocking for 1 hour in 5% nonfat milk dissolved in TBS with 0.1% Tween-20, membranes were incubated overnight at 4°C with primary antibodies ( Supplementary Table S2 , available in the online version only). Membranes were then incubated with HRP-conjugated anti-rabbit or anti-mouse IgG for 1 hour at room temperature. The immunoreactive bands were detected by chemiluminescence (Western ECL substrate, Bio-Rad) using a luminescent image analyzer system (LAS-4000 mini, Fujifilm).

### Reactive Oxygen Species Production

Measurement of oxidant level in SMCs was performed using the fluorescence-labeled probe 5- and 6-chloromethyl-2,7′-dichlorodihydrofluorescein diacetate, acetyl ester (CM-H _2_ DCFDA; Life Technologies). SMCs were seeded at a density of 1 × 10 ^4^ cells/well in a 96-well flat-bottom clear, black polystyrene plate (Corning) and starved overnight. After washing with Hank's balanced salt solution, SMCs were incubated for 1 hour with 5 µM CM-H _2_ DCFDA diluted in Opti-MEM. Fluorescence intensity was measured using a 480 nm excitation and a 530 nm emission filter set.

### Cellular Metabolism

Mitochondrial respiration assessed by oxygen consumption rate was determined with the Seahorse XFp Analyzer and the Cell Mito Stress Test kit (Agilent Technologies Inc., Santa Clara, California, United States). SMCs were seeded overnight on XFp cell culture miniplates (eight wells) at 5,000 cells per well in Dulbecco's modified Eagle's medium (DMEM) supplemented with 2 mM L-glutamine and 10% fetal bovine serum. Three replicate miniplate wells were used for each type of SMCs. Measurements were performed in DMEM XF Assay media (unbuffered DMEM supplemented with 10 mM glucose, 2 mM L-glutamine, and 1 mM sodium pyruvate) in the presence of the following inhibitors: 1.5 µM oligomycin, 0.5 µM carbonyl cyanide-p-trifluoromethoxyphenylhydrazone (FCCP), and 0.5 µM rotenone/antimycin A to determine the basal respiration, ATP-linked respiration, proton leak respiration, maximal respiration capacity, and reserve capacity.

### Statistical Analysis

Data are presented as mean ± SD. Statistical analysis was performed using Graphpad Prism 7 software. Normality of distribution was verified before each analysis. One-way ANOVA followed by Tukey's multiple comparisons test was performed when comparing multiple groups, and the Mann-Whitney test was used for the comparison of two groups. A *p* -value < 0.05 was considered significant.

## Results

### Prothrombin and Fibrinogen Were Present in AAA and TAA Aortic Tissues, But Fibrin Only in AAA

**Fig. 1:**
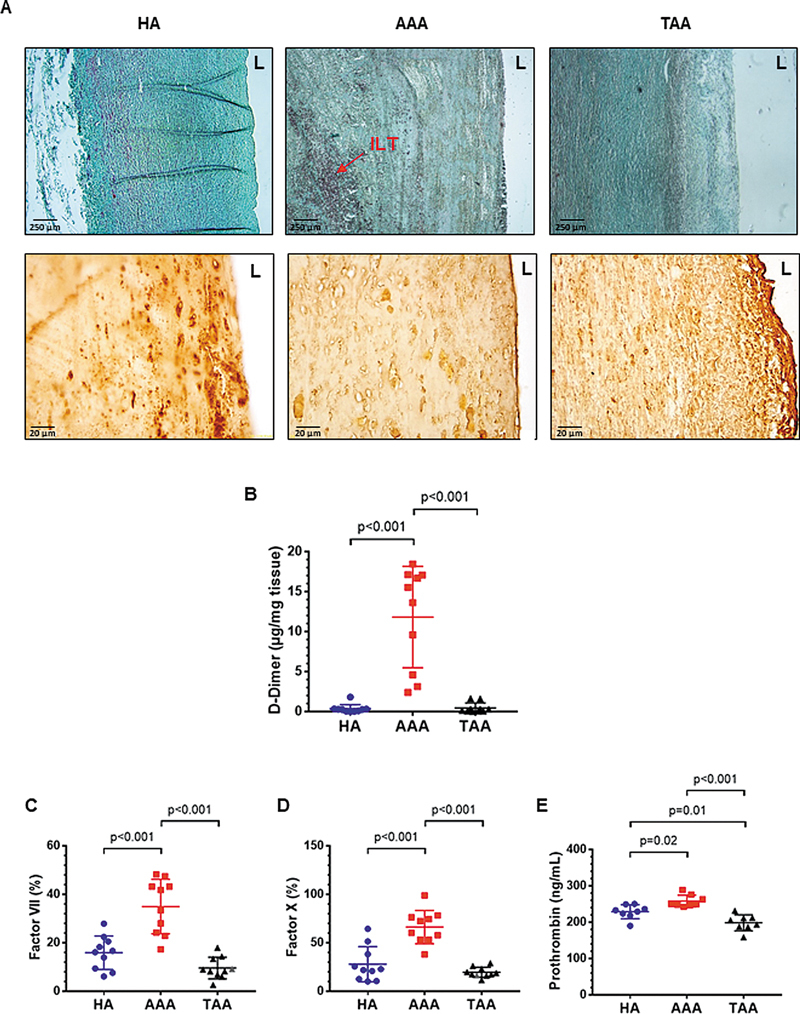
Fibrinogen deposition in aortic tissues in aneurysms and procoagulant state of conditioned media of aortic tissues. ( **A** ) Masson's trichrome staining (upper panels) and immunostaining for fibrinogen (lower panels) in the aorta from healthy donors (HA), abdominal aortic aneurysm (AAA), and thoracic aortic aneurysm (TAA). ILT: intraluminal thrombus. ( **B** ) D-dimer concentration in the conditioned media of aortic tissues. ( **C–E** ) Factor VII, factor X, and prothrombin concentration in conditioned media from HA, AAA, and TAA aortas. Data are presented as the means ± SD. Groups were analyzed statistically by a one-way ANOVA test and a Tukey test for multiple comparisons ( *n* = 8–10).

To explore the potential implication of SMCs in thrombus formation in AAA, we first observed the aortic tissues of patients with AAA or TAA as compared with HA for the presence of fibrinogen and fibrin ( Fig. 1 ). Masson's trichrome staining confirmed the presence of ILT in AAA wall but not in TAA wall ( Fig. 1A ). As expected, fibrinogen was observed in the three aortic types ( Fig. 1A ). There was a gradient of fibrinogen expression from the luminal layer to the adventitia in the HA and TAA wall, suggesting that tissue fibrinogen was mainly outwardly convected from the plasma. The presence of intact fibrinogen was less pronounced in the AAA wall, suggesting that fibrinogen may have been consumed to produce fibrin. Confirming this hypothesis, active fibrinoformation was evaluated by D-dimer release in the tissue-conditioned media of aortic walls ( Fig. 1B ). D-dimer release was higher in the AAA wall, but lower in tissue-conditioned media of TAA and HA wall tissue ( *p* < 0.001).

To determine the thrombin-generating potential of aortic tissues, we then examined the content of FVII, FX, and prothrombin of tissue-conditioned media of the aorta from healthy subjects or patients with AAA. Tissue-conditioned media from AAA had significantly higher levels of these three key coagulation proteins of the extrinsic coagulation pathway, compared with tissue-conditioned media from healthy media or TAA ( Fig. 1C–E ).

### Thrombin Generation was Increased at the Surface of SMCs from AAA and Decreased at the Surface of SMCs from Patients with TAA

**Fig. 2:**
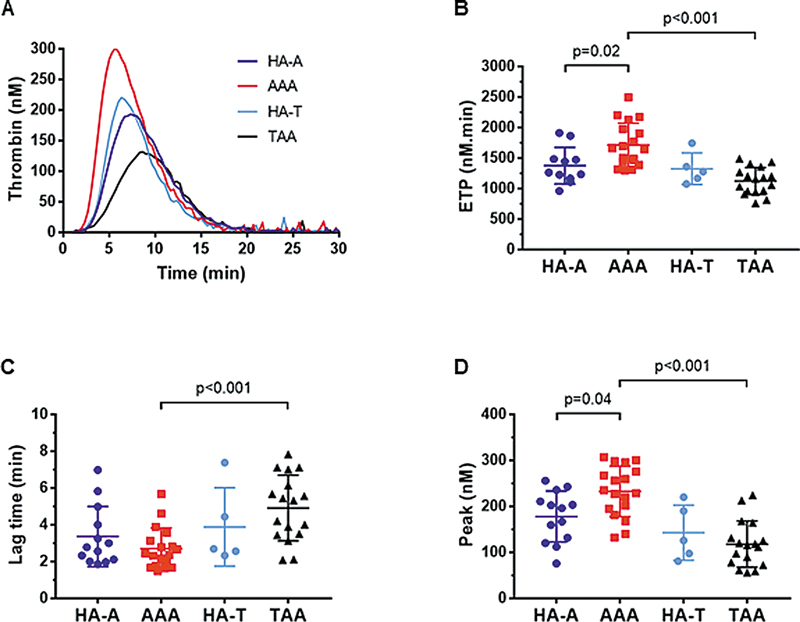
Thrombin generation at the surface of SMCs cultured from HA, AAA, or TAA. ( **A** ) Representative thrombin generation curves at the surface of SMCs cultured from HA, AAA, and TAA aortas. ( **B** ) Endogenous thrombin potential (ETP) values are calculated as the area under the curves of thrombin generation. ( **C** ) Lag-time derived from thrombin generation curves. ( **D** ) Thrombin peak derived from thrombin generation curves. Data are presented as the means ± SD. Groups were analyzed statistically by a one-way ANOVA test and a Tukey test for multiple comparisons ( *n* = 26–28).

To examine the contribution of SMCs in the thrombin-generating potential of aortic tissues, we monitored thrombin generation at the surface of cultured SMC monolayers. Thrombin formation did not occur in the absence of plasma or in the presence of factor VII or X-deficient plasma. In the presence of prothrombin-deficient plasma, similar low levels of thrombin (approximately 1 nM) were formed at the surface of all three types of SMCs (data not shown). To further investigate the effect of aneurysm on thrombin generation at the surface of SMCs, the same pooled human plasma from healthy donors was added to the three types of adherent SMCs. Given a sex distribution in the group of HA different from that of aneurysmal aortas, we first sought to confirm the absence of sex differences in the ability of HA-derived SMCs to support thrombin generation. Thrombin generation curves and ETP values did not differ between the sexes ( Supplementary Fig. S1 , available in the online version only). Typical curves at the surface of SMCs in the presence of pooled normal plasma show that thrombin generation was higher for AAA-derived SMCs compared with HA-derived SMCs of the same origin and to TAA-derived SMCs ( Fig. 2A ). In the absence of exogenous TF, the total thrombin activity assessed by ETP values was significantly higher at the surface of SMCs from AAA and lower from TAA when compared with SMCs from HA ( Fig. 2B ). Whereas no difference was observed in lag time for AAA-derived SMCs and HA-derived SMCs, lag time was prolonged at the surface of TAA-derived SMCs ( Fig. 2C ). In agreement with the ETP differences, the thrombin peak was increased at the surface of AAA-derived SMCs and decreased at the surface of TAA-derived SMCs compared with those at the surface of HA-derived SMCs ( Fig. 2D ). Similar ETP values were observed at different cell passages, with persistent elevated values at the surface of AAA-derived SMCs and lower values at the surface of TAA-derived SMCs ( Supplementary Fig. S2 , available in the online version only). Together, these results suggest that SMCs contribute to an increased prothrombotic state of the AAA wall and to an opposite antithrombotic profile of the TAA wall.

### PS Exposure and Thrombin-Dependent PAR-1 Signaling Enhanced Thrombin Generation at the Surface of SMCs from Patients with AAA

**Fig. 3:**
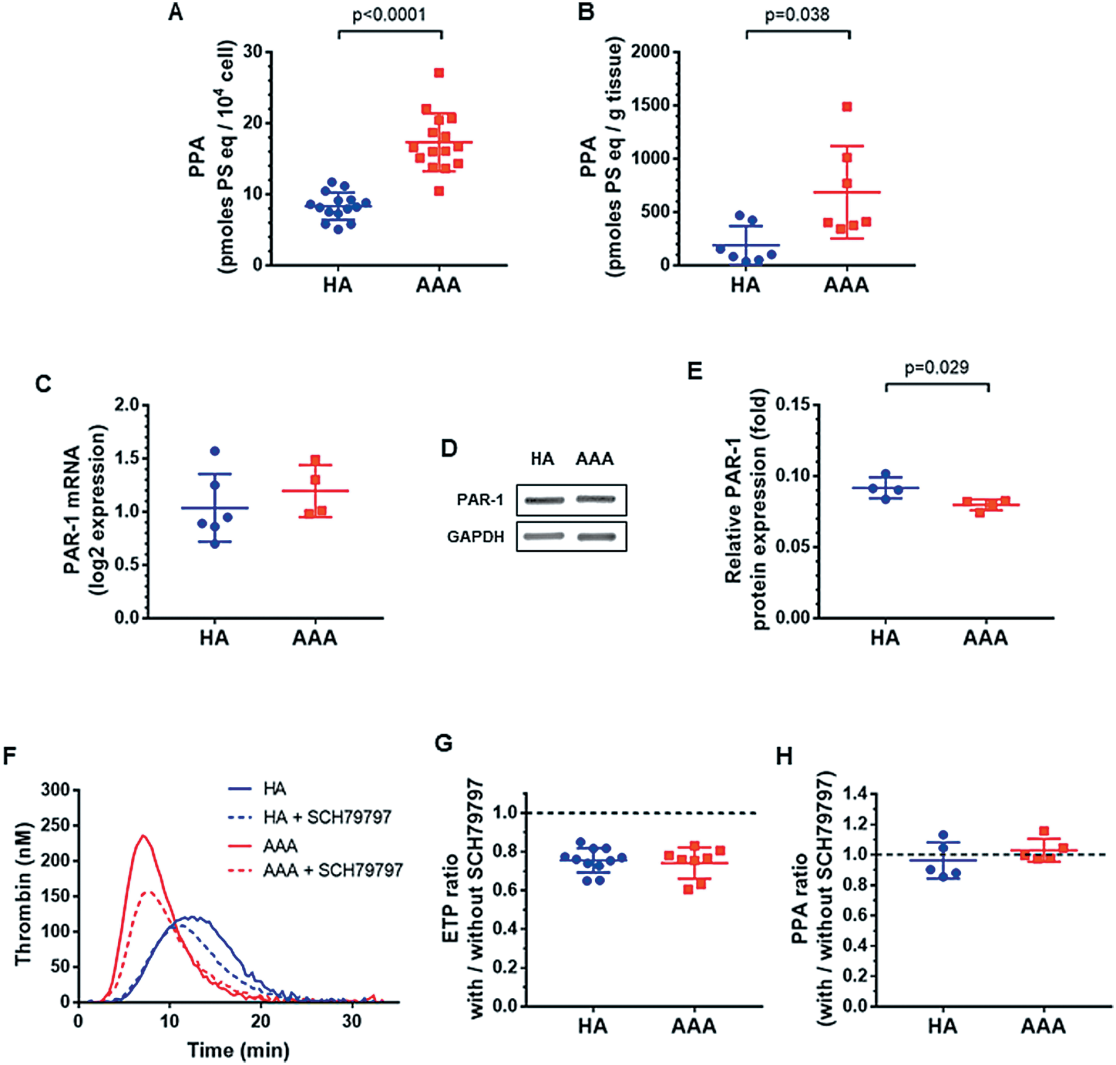
Exposure of anionic phospholipids and contribution of PAR-1 activation in thrombin generation at the surface of SMCs from HA and AAA. ( **A** ) Phospholipid procoagulant activity (PPA) at the surface of SMCs ( *n* = 15). ( **B** ) Phospholipid procoagulant activity in tissue-conditioned media ( *n* = 7–8). ( **C** ) PAR-1 synthesis by SMCs cultured from HA and AAA aortas ( *n* = 4–6). ( **D** ) Representative images of Western blot to assess levels of PAR-1 in SMCs. ( **E** ) Quantification of PAR-1 band intensities ( *n* = 4). ( **F** ) Representative thrombin generation curves at the surface of SMCs preincubated or not for 1 hour with the PAR-1 antagonist SCH79797. ( **G** ) Thrombin generating capacity (ETP) in the presence of SCH79797 normalized by the ETP value in the absence of SCH79797 ( *n* = 8–11). ( **H** ) Phospholipid procoagulant activity at the surface of SMCs incubated for 1 hour with SCH79797, normalized by PPA value in the absence of SCH79797 ( *n* = 5). Data are presented as the means ± SD. Groups were statistically analyzed by the Mann–Whitney test.

We next investigated the mechanisms underlying the procoagulant profile of SMCs from AAA. Given the critical role of negatively charged membrane phospholipids in the assembly of tenase (factors IXa/VIIIa/X) and prothrombinase (factors Xa/Va/II) complexes, we explored their exposure at the surface of SMCs and their release into tissue-conditioned media. PS exposure was approximately twofold higher at the surface of AAA-derived SMCs compared with HA-derived SMCs ( Fig. 3A ). The phospholipid procoagulant activity of AAA tissue-conditioned media was also significantly higher than in HA tissue-conditioned media ( Fig. 3B ). Because thrombin-PAR signaling has been shown to accelerate TF-induced thrombin generation, we next sought to determine the effect of PAR-1 inhibition on thrombin generation and PS exposure. Both HA-derived and AAA-derived SMCs synthesized and expressed similar levels of PAR-1 ( Fig. 3C–E ). Antagonism of PAR-1 with a specific peptide (SCH79797) reduced the thrombin peak at the surface of HA-derived and AAA-derived SMCs ( Fig. 3F, G ). Pretreatment with SCH79797 had no effect on PS exposure at the surface of SMCs ( Fig. 3H ). Collectively, these findings suggest that the increased thrombin generation in AAA was due, at least in part, to an increase in anionic phospholipids but not to enhanced PAR-1 activation.

### Activation of the PAR-2–NF-kB Axis in SMCs from AAA Regulated TF Activity

**Fig. 4:**
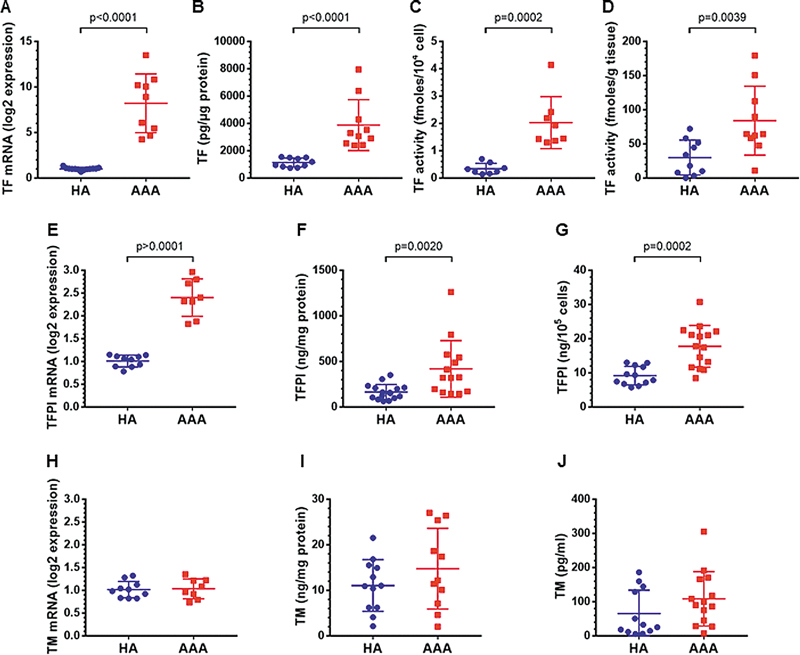
Expression of tissue factor and anticoagulant proteins in AAA. ( **A** ) Tissue factor (TF) synthesis by SMCs cultured from HA and AAA aortas ( *n* = 9–13). ( **B** ) TF antigen in SMCs ( *n* = 10). ( **C** ) TF activity at the surface of SMCs ( *n* = 8). ( **D** ) TF activity in tissue-conditioned media from HA and AAA ( *n* = 10). ( **E** ) Tissue factor pathway inhibitor (TFPI) mRNA levels in SMCs cultured from HA and AAA aortas ( *n* = 8–10). ( **F** ) TFPI protein in SMCs ( *n* = 14–16). ( **G** ) TFPI protein in cell supernatants ( *n* = 12–15). ( **H** ) Thrombomodulin (TM) mRNA levels in SMCs cultured from HA and AAA aortas ( *n* = 8–10). ( **I** ) TM protein in SMCs ( *n* = 11–12). ( **J** ) TM protein in cell supernatants ( *n* = 12–14). Data represent means ± SD. Groups were statistically analyzed by the Mann–Whitney test.

To provide mechanistic insight into the prothrombotic potential of AAA-SMCs, we assessed the expression and activity of TF, the main trigger of the extrinsic coagulation pathway. Gene expression, antigen expression, and procoagulant activity of cell-expressed TF were markedly increased in AAA-SMCs ( Fig. 4A–C ) compared with HA-SMCs. In addition, TF activity was also increased in AAA tissue-conditioned media ( Fig. 4D ), thus consolidating that TF is a key player in the prothrombotic state of the AAA wall. To evaluate the procoagulant and anticoagulant balance at the surface of AAA-SMCs, expression of TFPI and thrombomodulin was determined. Cell-expressed TFPI as well as TFPI released in cell supernatant were increased in AAA-SMCs ( Fig. 4E–G ), while no change in thrombomodulin expression and release was observed ( Fig. 4H–J ).

**Fig. 5:**
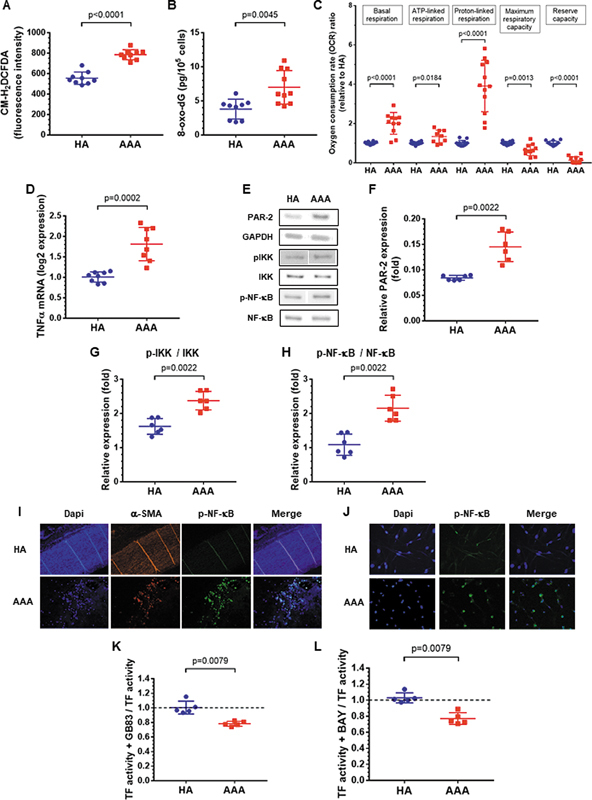
Involvement of the PAR-2/NF-κB axis in increased tissue factor expression in AAA. ( **A** ) Fluorescent detection of cellular oxidant levels using CM-H _2_ DCFDA ( *n* = 9). ( **B** ) Levels of 8-oxo-7,8-dihydro-2′-deoxyguanosine (8-oxo-dG) in cell supernatants ( *n* = 9–10). ( **C** ) Seahorse respiratory measurements, namely basal respiration, ATP-linked respiration, proton-linked respiration, maximum respiratory capacity, and reserve capacity in AAA-SMCs compared with HA-SMCs ( *n* = 9–12). ( **D** ) TNF-α mRNA levels in SMCs cultured from HA and AAA aortas ( *n* = 8). ( **E** ) Representative images of Western blot to assess levels of PAR-2, phosphorylated and total IKK, and NF-κB in SMCs. ( **F–H** ) Quantification of PAR-2, p-IKK/IKK, and p-NF-κB/NF-κB band intensities ( *n* = 5–6). ( **I** ) Immunofluorescence staining of α-SMA (red) and p-NF-κB (green) in aortic tissues. Nuclei were counterstained using DAPI. ( **J** ) Immunofluorescence staining of p-NF-κB in SMCs. ( **K, L** ) TF activity at the surface of SMCs incubated for 4 hours with the PAR-2 antagonist GB83 or the inhibitor of NF-κB activation BAY 11–7082 (BAY). Values are normalized by TF activity in the absence of GB83 or BAY ( *n* = 5). Data represent means ± SD. Groups were statistically analyzed by the Mann–Whitney test.

Since reactive oxygen species (ROS), which are activators of the NF-κB pathway, can stimulate TF expression, we assessed the presence of ROS in AAA-SMCs. Levels of the fluorogenic probe CM-H _2_ DCFDA, as well as extracellular 8-oxo-dG, a marker of oxidative DNA damage, were increased in AAA-SMCs ( Fig. 5A, B ). Consistent with these findings, AAA-SMCs displayed worsened mitochondrial function ( Fig. 5C ; Supplementary Fig. S3 , available in the online version only). Basal respiration was significantly higher for AAA-SMCs. Both ATP-linked respiration and proton-linked respiration, which are the two components of basal respiration, are increased in AAA-SMCs. Maximal respiratory capacity was overall significantly decreased, resulting in a significantly reduced reserve capacity in AAA-SMCs.

We next analyzed whether the NF-κB pathway via PAR-2 signaling contributed to the transcriptional induction of TF in AAA-SMCs. In addition to ROS, TNF-α is a strong inducer of PAR-2 expression. TNF-α mRNA was significantly increased in AAA-SMCs ( Fig. 5D ). As expected, AAA-SMCs exhibited higher expression of PAR-2 compared with HA-SMCs ( Fig. 5E, F ). Activation of the canonical NF-κB pathway involves phosphorylation of IKKa/b, whose degradation via the proteasomal pathway signals nuclear translocation of the downstream NF-κB p50/p65 heterodimer. Therefore, we tested the levels of phosphorylation of IKKa/b at the Ser176/180 site and the levels of phosphorylation of NF-κB at the Ser536 site. Both phosphorylated IKKa/b and the NF-κB p65 subunit were increased in AAA-SMCs compared with HA-SMCs ( Fig. 5G, H ). Double immunofluorescence staining for phosphorylated p65 and smooth muscle actin revealed that the expression of phosphorylated p65 was detected only in AAA and was associated with medial SMCs ( Fig. 5I ). Further, nuclear translocation of p65 was also shown in AAA-SMCs ( Fig. 5J ), indicating that NF-κB triggers transcription of its target genes, including TF in AAA. Incubation of SMCs with the selective PAR-1 antagonist (SCH79797) reduced FT activity, but with no significant difference between AAA-SMCs and HA-SMCs ( Supplementary Fig. S4 , available in the online version only). Incubation of SMCs with the selective PAR-2 antagonist GB83 or BAY, that inhibits NF-κB activation significantly reduced TF activity at the surface of AAA-SMCs without any effect on TF activity at the surface of HA-SMCs ( Fig. 5K, L ). Together, these data support PAR-2–NF-κB axis activation that is required for increased TF activity at the surface of AAA-SMCs, which in turn enhanced thrombin generation in AAA.

## Discussion

Our findings demonstrate a procoagulant phenotype of AAA-derived arterial media and SMC cultures compared with HA and TAA in humans. To date, coagulation changes in AAA have been studied mainly in endothelial cells and plasma. Here, we have focused on SMCs, which are known to be prothrombotic and therefore potentially responsible for ILT formation. We observed a high degree of consistency between the results observed at the tissue level and those observed in SMC cultures. The mechanisms involve an increase in cell surface expression of PS and a PAR-2-induced NF-κB-mediated overexpression of TF in AAA-derived SMCs.

In a first set of tissue experiments, we show that plasma fibrinogen penetrates the arterial wall in both AAA, HA, and TAA. Like all plasma proteins, plasma fibrinogen is outwardly convected through the wall, where it can be converted into fibrin if all the players of the coagulation cascade are present in close proximity. As previously described in advanced atherosclerosis, 17 the absence of a functional endothelial barrier in AAA is expected to promote the accumulation of plasma clotting factors involved in the TF-dependent coagulation pathway within the vessel wall and the subsequent dynamic process of fibrin formation and degradation. The findings that factor VII, factor X, and prothrombin concentrations were significantly increased in AAA tissue-conditioned media as compared with HA and TAA are consistent with such an accumulation of plasma clotting factors that favors thrombin generation induced by SMC-expressed TF. Our study showed a fibrinogen gradient across the healthy arterial wall. The absence of such a gradient in the AAA wall suggests that fibrinogen has been converted to fibrin via thrombin-mediated proteolytic cleavage and that secondary fibrinolysis has occurred, generating D-dimer released in the AAA tissue-conditioned media. D-dimer is a well-known biomarker of coagulation activation and secondary fibrinolysis, in agreement with our previous report on the topology and activity of the fibrinolytic system in human AAA lesions. 18 This argues for greater intravascular fibrin turnover in AAA as compared with HA and TAA.

It has been well documented that SMCs are the main sources of TF in the arterial wall. 19 20 The occurrence of a thrombin burst on the surface of SMCs in the absence of exogenous TF in our assay is consistent with previous studies showing that addition of TF is not required to trigger thrombin formation on the surface of arterial tissue. 12 While factor VII and factor X/Xa, colocalizing with TF, have been previously detected on the cell surface in SMCs in human atherosclerotic vessels, 17 the addition of factor VII or factor X-deficient plasma to cultured SMCs from HA, AAA, and TAA did not elicit thrombin formation. However, traces of thrombin are generated in the presence of prothrombin-deficient plasma, which is consistent with the reported detection of prothrombin in SMCs in atheroma and in TAA aortic sections. 17 21 In the presence of normal plasma, TF-initiated thrombin generation, assessed by ETP and thrombin peak values, was significantly enhanced at the surface of AAA-SMCs as compared with HA-SMCs. In contrast, thrombin generation was decreased at the surface of TAA-SMCs. We have previously demonstrated that protease nexin-1, which is produced and secreted by SMCs in the arterial wall, is overexpressed at both the mRNA and protein levels in TAA aortic tissues and SMCs. 15 22 This serpin is known to be a powerful inhibitor of thrombin activation, both in in vitro and in vivo experiments. 22 23 TF-induced activation of the coagulation is consistent with the significant increase in TF expression and activity in AAA-SMCs as compared with HA-SMCs. Aside from cell-associated TF, soluble TF circulates in plasma 24 and it has been reported that SMCs can release active TF. 20 Echoing the increase in cell-associated TF in AAA-SMCs, we found that TF activity released in tissue-conditioned media was increased in AAA as compared with HA, which is in line with previous studies showing higher levels of TF in both ILT and adjacent AAA walls, as well as in the plasma of patients with AAA. 25 26 So far, however, Scott et al 27 found slightly decreased TF-induced thrombin generation but in platelet-poor plasma of AAA patients compared with controls. In a recent proteomic study of AAA ILT and wall, prothrombin and other proteins of the coagulation and fibrinolytic cascade were identified in the wall, and their overexpression correlates with the corresponding AAA expansion rate and perpetuation. 1 Thus, our results clearly reflect the major role that SMCs could play in the formation of ILT in AAA. Mirroring the increase in TF, SMC-associated TFPI is increased in AAA-SMCs. However, our data suggest that this increase in the anticoagulant potential of AAA-SMCs is not sufficient to compensate for the increase in procoagulant potential at a later stage of AAA.

Exposure of negatively charged phospholipids together with expression of active TF on the cell surface are important events in thrombin generation. These negatively charged phospholipids at the surface of SMCs were held responsible for the prothrombotic phenotype in hypertension. 10 Surface expression of PS was detected on viable SMCs irrespective of their origin (HA or AAA), which agrees with the previous demonstration of the occurrence of thrombin generation on the surface of nonapoptotic SMCs. 12 PS exposure was increased in AAA-SMCs, resulting in enhanced membrane phospholipid procoagulant activity. In addition, we observed an increase in PS-containing surface in AAA tissue-conditioned media, concomitant with the rise in TF levels. These data support the assumption that vesicles containing externalized PS and TF may bud from the surface of SMCs in response to cell activation and represent a source of SMC-procoagulant activity. 20

Extracellular procoagulant signals are mainly transduced by the G-protein-coupled PARs. Thrombin-mediated signaling through PARs has been reported to increase PS-exposing cells and thereby to accelerate TF-initiated thrombin generation. 28 29 Activation of PAR-3 or PAR-4 but not PAR-1 has been reported to increase SMC-supported TF-initiated thrombin generation. We found similar PAR-1 expression (mRNA and protein) whatever the origin of SMCs (HA or AAA). Pharmacological antagonism of PAR-1 decreased thrombin generation to the same extent in HA- and AAA-SMCs. The discrepancy with the previous study of Vidwan et al 28 could be explained by differences in experimental conditions, as we tested the PAR-1 antagonist in a different thrombin generation assay and without stimulation of SMCs by exogenous thrombin. In an attempt to elucidate whether changes in PS exposure are responsible for the observed inhibition, we quantified SMC procoagulant activities. Our data showed that inhibition of PAR-1 failed to increase PS exposure in all SMCs. Thus, our results support the conclusion that PAR-1 is not involved in increased thrombin generation at the surface of AAA-SMCs compared with HA-SMCs.

Expression of TF was upregulated by numerous factors, including oxidative stress and the NF-κB pathway activation. 30 Oxidative stress through IKK phosphorylation and degradation allows p-NF-κB nuclear translocation and induction of gene expression. 31 Several studies have reported redox-sensitive TF induction mediated by PAR-1 and/or PAR-2 activation, inducing NF-κB nuclear translocation in endothelial cells. 32 33 It has been reported that an increase in TF expression can be paralleled by an increase in PAR-2 in vascular SMCs. 34 PAR-2, which is activated by the TF/activated factor VII complex or factor Xa, 35 has been shown to regulate TF gene expression through the NF-κB signaling pathway since the promoter of the human TF gene contains NF-κB binding sites. 34 36 Accordingly, at the tissue level, we did observe an activation of the NF-κB signaling pathway in the arterial wall of AAA as compared with HA. Once again, these results were reproduced in AAA-cultured SMCs. NF-κB nuclear translocation is associated with an increase in TNF-α and oxidative stress inherent to nuclear and/or mitochondrial DNA damage, which are consistent features of AAA, 37 38 as well as in PAR-2 expression in AAA-SMCs. Taken altogether, our data suggest that TF expression and activity are increased principally through a PAR-2/NF-κB signaling pathway. This assumption is supported by the finding that the inhibitors of PAR-2 (GB83) or NF-kB (BAY) were both able to significantly decrease TF expression in AAA-SMCs but did not impact TF expression in HA-derived SMCs. Therefore, we can conclude that a dynamic process leading to TF-mediated activation of the coagulation occurs in cultured SMCs from the AAA arterial wall origin.

The phenotype coherence between ex vivo tissues and primary SMC cultures, as well as the sustainability of the procoagulant state through numerous mitoses, turns the attention toward an epigenetic imprinting in AAA, as previously reported for tensile stress in TAA. 39 NF-κB dynamics determine epigenomic reprogramming in an environmental stimulus-specific manner. 40 41 While there may be several potential mechanisms involved in TF activation (decryption of TF due to the externalization of PS, exposure to lipids, allosteric conformational effect of oxidation, action of protein disulfide isomerase…), 42 43 our results suggest that the increase in TF activity could be the consequence of an increase in gene expression. Future investigations will need to uncover the role of epigenetic regulation of TF and the potential therapeutic benefit of epigenetic modifiers in fighting the prothrombotic state in the arterial wall.

In summary, our results demonstrate the contribution of NF-κB-mediated upregulation of TF in the procoagulant phenotype of AAA media and SMCs. An unresolved question is whether thrombin generation at the surface of AAA-SMCs is a trigger of ILT formation or, conversely, ILT modifies the procoagulant potential of SMCs. Nevertheless, our results point to a crucial role of SMCs in perpetuating a thrombogenic cycle in AAA tissue. While TF-based therapy may be unsafe due to the risk of bleeding, direct targeting of the TF coagulant function at the site of thrombosis in AAA could represent an alternative approach.

## Notes

**What is known about this topic?**

- Aneurysm of the abdominal aorta (AAA) is an acquired atherothrombotic disease, characterized at a late stage by the presence of an ILT.
- Due to the presence of the ILT, the endothelium of the AAA wall is highly damaged, exposing SMCs to blood cells and molecules.
- Peroxidases released from blood cells entrapped in the ILT foster hemolytic-dependent oxidative stress.

**What does this study add?**

- Human AAA-derived SMC cultures exhibit a higher production of TF compared with SMCs from healthy aorta.
- Tissue-factor upregulation increases thrombin generation at the surface of AAA-derived SMCs, perpetuating the thrombogenic cycle in the AAA wall.
- The mechanism involves PS exposure and PAR-2/NF-κB signaling.
