# Supplementary material for: NF-κB-Mediated Upregulation of Tissue Factor Contributes to the Procoagulant Phenotype of Smooth Muscle Cells from Abdominal Aorta Aneurysm in Human
Source: Thromb Haemost. 2025 Aug 12;126(3):319–31. doi: 10.1055/a-2665-2510 (PMC12932002; doi:10.1055/a-2665-2510)
Supplement: Supplementary file 1 — Supplementary Material [file 10-1055-a-2665-2510-s25030174.pdf]

Thieme

Supplementary material

Supplementary Table S1: demographic data of human tissue samples

HA				AAA			TAA		
**Code**	**Sex**	**Age**	**Origin**	**Code**	**Sex**	**Age**	**Code**	**Sex**	**Age**
1	F	87	AA	1	M	51	1	M	68
2	M	74	AA	2	M	65	2	M	32
3	M	50	AA	3	M	65	3	F	83
4	F	80	AA	4	M	50	4	M	71
5	F	68	TA	5	M	73	5	M	46
6	F	72	TA	6	M	62	6	M	61
7	F	35	TA	7	M	55	7	M	62
8	M	51	TA	8	M	71	8	F	63
9	F	67	TA	9	M	74	9	F	54
10	F	45	AA	10	M	81	10	M	57
11	F	79	AA	11	F	66	11	M	55
12	M	21	TA	12	M	65	12	M	68
13	M	23	AA	13	M	65	13	F	55
14	F	24	AA	14	F	69	14	M	50
15	F	67	AA	15	M	62	15	M	53
16	M	26	AA	16	M	74	16	M	41
17	M	52	AA	17	F	61	17	M	41
18	M	53	TA	18	F	73	18	M	55

Supplementary Table S2: Antibodies

Name	Vendor or Source	Clone or reference	Dilution
Rabbit polyclonal to human fibrinogen	Abcam	ab34269	1:100 IF
Mouse monoclonal antibody to a-Smooth Muscle Actin	Sigma Aldrich	A2547	1:300 IF
Rabbit monoclonal antibodies to human phospho-NF-κB p65 (Ser536)	Cell Signaling Technologies	3033	1:200 IF<br>1:1000 WB
Rabbit monoclonal antibodies to human NF-κB p65	Cell Signaling Technologies	8242	1:1000 WB
Mouse monoclonal antibody to human PAR-2	Santa Cruz	sc-13504	1:500 WB
Rabbit monoclonal antibodies to human phosphor-IKKα/β (Ser176/180)	Cell Signaling Technologies	2697	1:1000 WB
Rabbit monoclonal antibodies to human phosphor-IKKβ	Cell Signaling Technologies	8943	1:1000 WB
Rabbit polyclonal antibody to GAPDH	Santa Cruz	sc-25778	1:500 WB
Goat polyclonal antibodies to mouse IgG1, Alexa Fluor 555 conjugate	Invitrogen	A21127	1:1000 IF
Donkey polyclonal antibodies to rabbit IgG (H+L), Alexa Fluor 488 conjugate	Invitrogen	A21206	1:1000 IF

Supplementary Table S3: Primers

Gene	Primers	Sequence (5' → 3')
F3	Forward<br>Reverse	TGACCTCACCGACGAGATTGTGAA<br>TCTGAATTGTTGGCTGTCCGAGGT
TFPI	Forward<br>Reverse	ATTTCACGGTCCCTCATGGTGTCT<br>GGCGGCATTTCCCAATGACTGAAT
THBD	Forward<br>Reverse	TAACGAAGACACAGACTGCATT<br>CTAGCCCACGAGGTCAAGGT
TNF	Forward<br>Reverse	AGAGGGCCTGTACCTCATCTACTC<br>GGTTGACCTTGGTCTGGTAGGA
RPS29	Forward<br>Reverse	CAGCCTCAAGATCATCAGCA<br>TGTGGTCATGAGTCCTTCCA
GAPDH	Forward<br>Reverse	AAGATGGGTCACCAGCAGCTGTACTG<br>AGACACGACAAGAGCGAGAA

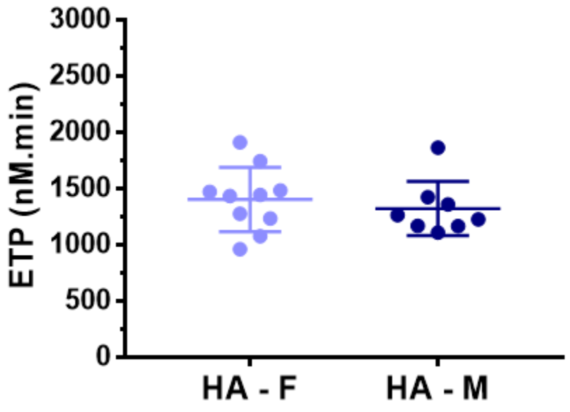

Supplementary Figure S1. Effect of sex on thrombin generation at the surface of SMCs cultured from HA. Endogenous thrombin potential (ETP) values calculated as area under the curves of thrombin generation as a function of female (F) or male (M) sex.

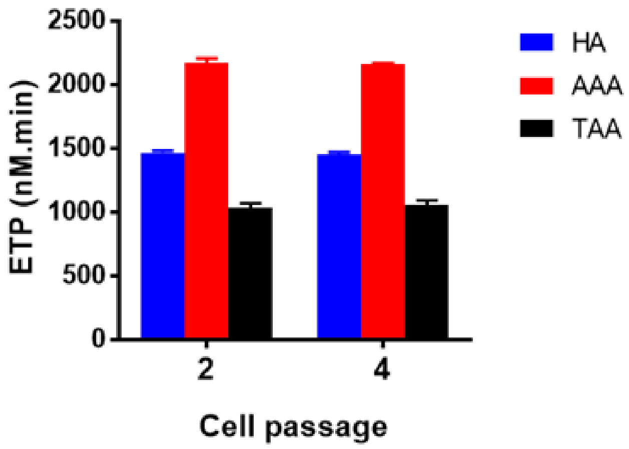

Supplementary Figure S2. Effect of cell passage on thrombin generation at the surface of SMCs cultured from SMCs cultured from HA, AAA or TAA. Endogenous thrombin potential (ETP) values calculated as area under the curves of thrombin generation at different cell passages.

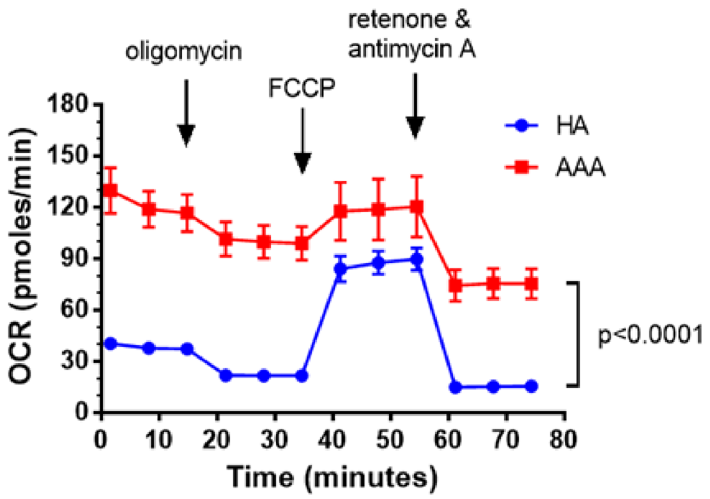

Supplementary Figure S3. Bioenergetic profile of SMCs cultured from HA or AAA. Oxygen consumption rate in SMCs before and after treatment with 1.5 µM oligomycin, 0.5 µM carbonyl cyanide-p-trifluoromethoxyphenylhydrazone (FCCP) and 0.5 µM rotenone/antimycin A.

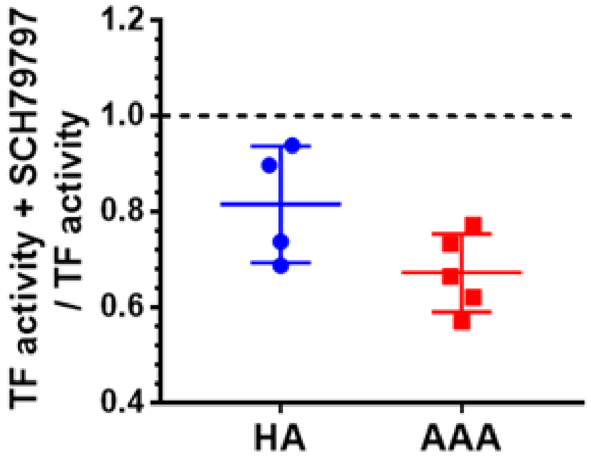

Supplementary Figure S4. Involvement of PAR-1 in increased tissue factor expression in AAA. TF activity at the surface of SMCs incubated for 1 hour with the PAR-1 antagonist SCH79797. Values are normalized by TF activity in the absence SCH79797 (n=4-5).

Thrombosis and Haemostasis | DOI 10.1055/a-2665-2510 | © The Author(s).
